# Di-Cavitary Twin Pregnancy in Didelphys Uterus with Associated Renal Agenesis

**DOI:** 10.1055/a-2562-1607

**Published:** 2025-04-10

**Authors:** Helen Woolcock Martinez, Noora Haghighi, Whitney A. Booker

**Affiliations:** 1 Division of Maternal-Fetal Medicine, Department of Obstetrics and Gynecology, Columbia University Irving Medical Center, New York, New York

**Keywords:** twin pregnancy, uterus didelphys, Müllerian duct anomaly, renal agenesis

## Abstract

**Introduction:**

A didelphys uterus is a rare Müllerian duct anomaly (MDA) caused by the incomplete fusion of the Müllerian ducts, leading to the formation of two distinct uterine cavities. The occurrence of simultaneous twin pregnancies, with each fetus developing in a separate horn of a didelphys uterus, is estimated at approximately 1 in 1,000,000 cases. This case report describes a rare instance of a spontaneous dichorionic diamniotic twin pregnancy, with one fetus in each horn of a didelphys uterus.

**Case Description:**

This is the case of a 35-year-old woman with unilateral renal agenesis who presented with a spontaneous dichorionic diamniotic twin pregnancy, with one fetus in each uterine cavity. This patient's pregnancy complications included intrahepatic cholestasis of pregnancy and rising creatinine levels, leading to a planned cesarean delivery at 36 weeks. The delivery was complicated by intrapartum hemorrhage and the postpartum course was further complicated by sepsis and endometritis.

**Conclusion:**

This case highlights the complexity of managing a dichorionic diamniotic twin pregnancy in a didelphys uterus with concomitant congenital unilateral renal agenesis. It contributes to the understanding of optimal management strategies for MDA presentations and highlights the necessity for ongoing research into complications and long-term outcomes associated with such anomalies.

## Introduction

A didelphys uterus is a developmental anomaly characterized by incomplete fusion of the Müllerian ducts, leading to the formation of two distinct uterine cavities. A didelphys uterus is one of the least common Müllerian duct anomalies (MDAs), comprising approximately 10% of all MDAs. 1 Simultaneous twin gestation with each fetus in a separate horn of a didelphys uterus is extremely rare, with a reported incidence of approximately 1 in 1,000,000. 2 Additionally, because of the interrelated development between the two tracts, MDAs can be associated with Wolffian duct abnormalities, often resulting in abnormalities of the kidneys and urinary tract. 3 In general, uterine malformations are associated with a higher risk of miscarriage, preterm labor, breech delivery, and decreased live births. Additionally, MDAs can impact an individual's fertility. 4 However, the extent of these outcomes depends on the type of MDA. We are discussing a rare case of a spontaneous twin pregnancy developed in each horn of a didelphys uterus.

## Patient Description

This patient is a 35-year-old G4P1112 with a medical history significant for unilateral renal agenesis who was referred to the maternal–fetal medicine service after presenting with a spontaneous dichorionic diamniotic twin pregnancy in the setting of a didelphys uterus, with a pregnancy in each uterine horn.

## Medical and Obstetric History

The patient's first pregnancy in 2011 was complicated by a preterm cesarean delivery for breech presentation at 36 ^6/7^ weeks gestational age. The infant was born at 1,871 g, requiring a 3-week stay in the neonatal intensive care unit. A didelphys uterus was noted at the time of cesarean, with the pregnancy in the right uterine horn. Each uterine horn had one fallopian tube, and one ovary attached. The second pregnancy in 2018 was characterized by a monochorionic diamniotic twin pregnancy in the left uterus. This pregnancy was complicated by unequal placental sharing and fetal growth restriction, resulting in dual fetal demise at 20 weeks gestational age. The patient underwent dilation and evacuation, which was further complicated by cervical and uterine perforation, requiring a blood transfusion. The third pregnancy in 2021 was a spontaneous singleton pregnancy in the left uterus. The patient delivered via repeat cesarean section for breech presentation at 37 weeks gestation.

This patient's medical history also includes right renal agenesis, which was diagnosed in 2013 and confirmed by a computed tomography (CT) scan in 2017. Her baseline creatinine at the first-trimester initial prenatal visit was 0.67 mg/dL with a repeat third-trimester creatinine of 0.83 mg/dL.

## Physical Exam and Ultrasonographic Investigations

**Fig. 1:**
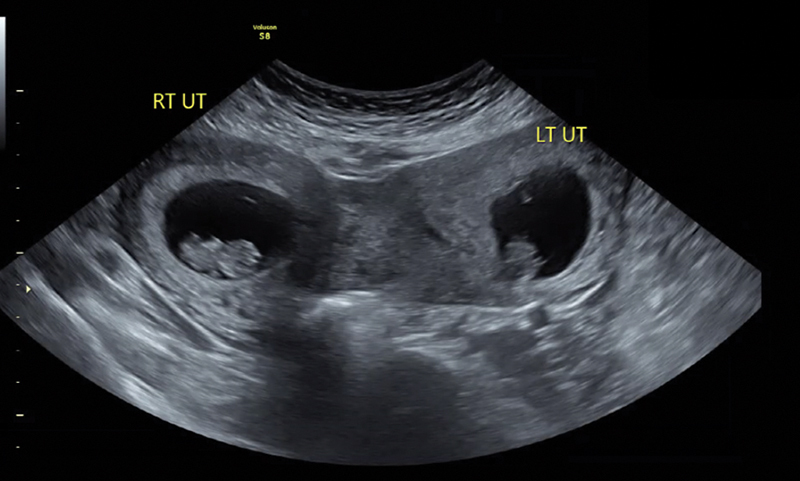
Transabdominal transverse view of the viability scan at 9 ^0/7^ weeks gestational age. LT UT, left uterus with twin A; RT UT, right uterus with twin B.

**Fig. 2:**
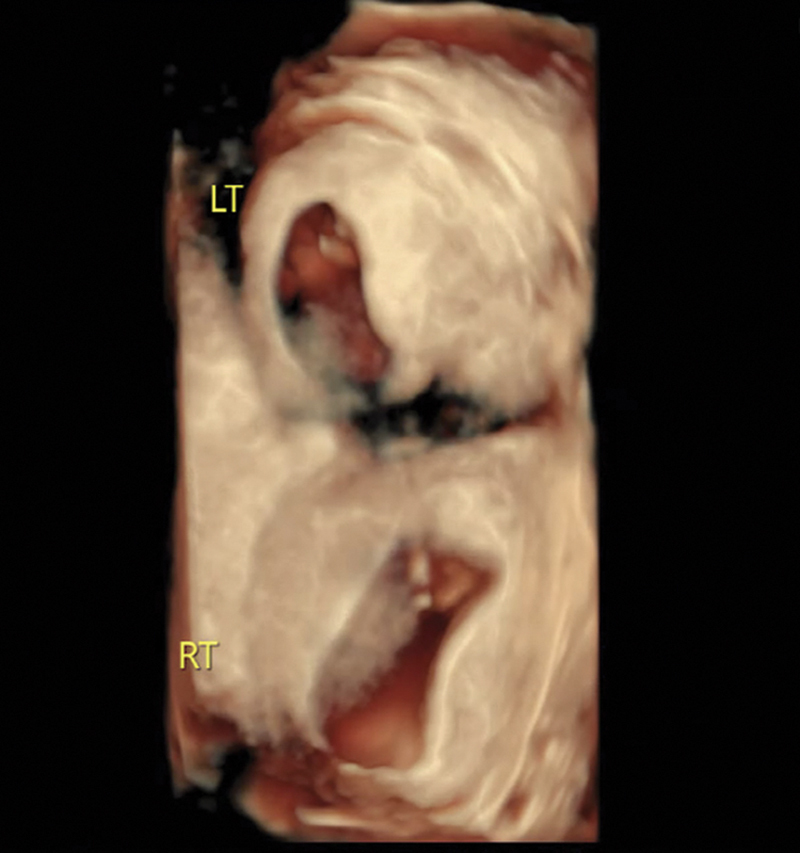
Three-dimensional rendering ultrasound at 11 ^1/7^ weeks gestational age. LT, left; RT, right.

An initial viability ultrasound was performed at 9 ^0/7^ weeks gestational age via transvaginal ultrasound by maternal–fetal medicine specialists. The ultrasound confirmed a didelphys uterus with a live intrauterine twin gestation, with one fetus in each uterine horn and two cervices ( Figs. 1 and 2 ). Twin A was in the left uterus and twin B was in the right uterus. A sterile pelvic exam confirmed two cervices with a vaginal septum. Blood pressure monitoring and urine analyses were performed at each prenatal visit. The patient also monitored her blood pressure at home throughout the duration of her pregnancy.

Routine aneuploidy screening with cell-free DNA was completed and revealed a low risk for trisomy 13, 18, and 21. A fetal anatomical survey was completed at 20 weeks and was found to be normal within the resolution restrictions of ultrasound. The presentation of the twins was assessed at each ultrasound and they remained cephalic-cephalic from 30 weeks gestational age until delivery.

**Fig. 3:**
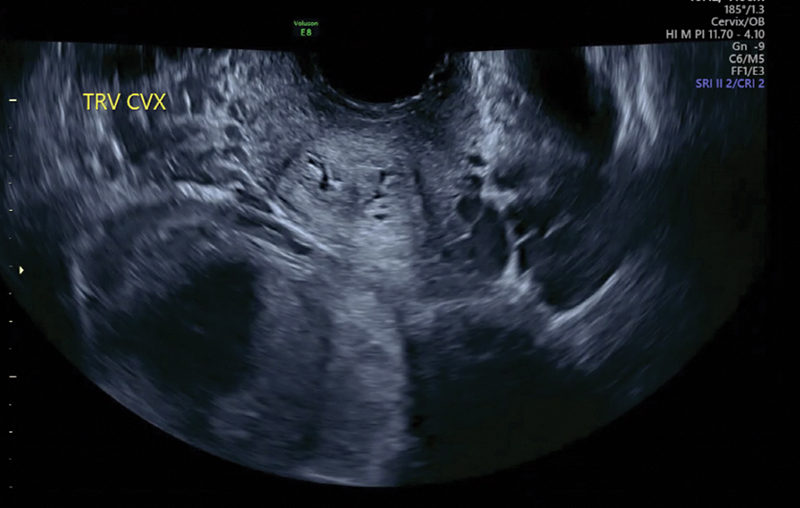
Transvaginal ultrasound at 18 ^3/7^ weeks gestational age demonstrating a double cervix in transverse view.

Serial ultrasonographic cervical length monitoring began at 18 weeks gestational age ( Fig. 3 ) and was performed every 2 weeks until 22 weeks gestational age, with the right cervical length stable between 4.2 and 4.7 cm and the left cervical length stable between 4.15 and 4.6 cm. Additionally, growth scans began at 22 weeks and were repeated every 3 weeks until 34 weeks gestational age. Both twins measured appropriately for their gestational age throughout the duration of the pregnancy. Placentae were located in the right lateral uterine wall in each uterine cavity by the time of delivery.

Fetal testing (biophysical profile [BPP]) began at 28 weeks gestational age and continued every 1 to 2 weeks until 34 weeks gestational age. At 25 weeks gestational age, the patient was found to have anemia of pregnancy, with a hematocrit of 30.7%, requiring three doses of intravenous iron infusion. At 33 weeks, the patient presented with pruritis and elevated bile acids, consistent with a diagnosis of intrahepatic cholestasis of pregnancy (ICP). In the setting of this new diagnosis, she was started on ursodiol (300 mg three times daily) and antepartum testing with BPPs was increased to twice weekly until delivery. At 35 weeks gestational age, she presented with left flank pain and an elevated creatinine to 1.22 from 0.83 mg/dL the month prior. Her blood pressure remained normal and there was low clinical suspicion for preeclampsia.

## Treatment

The initial delivery plan was a tertiary cesarean section at 38 weeks gestational age, given the patient's history of two prior cesarean sections. However, the delivery plan changed to 36 weeks in the setting of ICP with dichorionic diamniotic twins. She received 1 dose of betamethasone prior to delivery in the setting of preterm delivery. 5

The patient also desired bilateral tubal ligation at the time of the cesarean section. She was counseled on the risks of the procedure and consented days prior to the surgery.

### Cesarean Surgical Details

A Pfannenstiel skin incision was made with a scalpel at the prior incision site. The fascia was incised and extended laterally, the rectus muscles were separated, and the peritoneum was dissected, allowing for adequate visualization of the pelvic anatomy. Attention was then turned to the right uterine corpus, where a low transverse hysterotomy was made allowing for delivery of twin B. Subsequently, on the left uterine corpus, a vertical hysterotomy was made to deliver twin A.

**Fig. 4:**
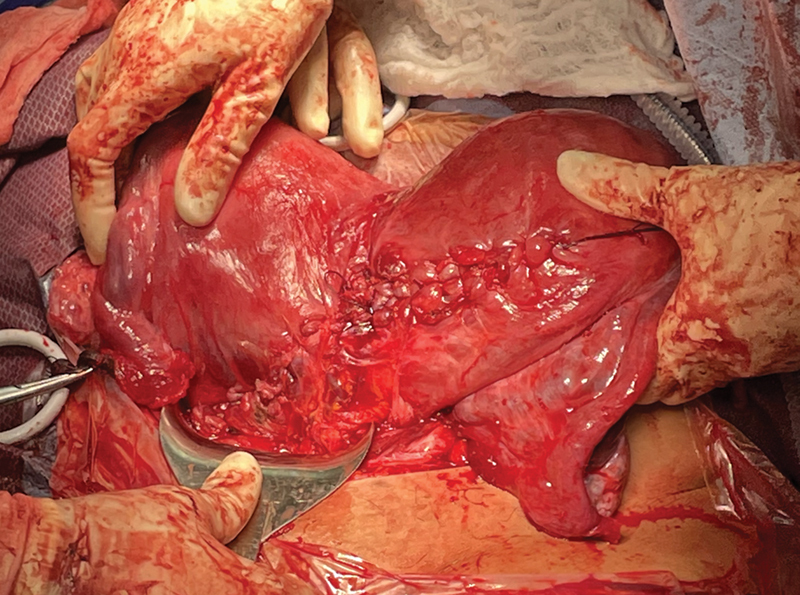
Exteriorized didelphys uterus with repaired vertical and transverse hysterotomies.

The uterus was exteriorized and cleared of all clots and debris. A 4 cm inferior extension of the hysterotomy was noted at the right aspect of the right hysterotomy. Significant bleeding was noted from the edges of the hysterotomies. Both hysterotomies were repaired in a running-locked fashion and a second imbricating layer was made to ensure hemostasis ( Fig. 4 ).

Due to uterine atony and increased bleeding at the hysterotomy sites, methergine, carboprost tromethamine, tranexamic acid, and 1 unit of packed red blood cells were given. After hemostasis was ensured and normal adnexal anatomy was confirmed, a bilateral salpingectomy was performed using a LigaSure device. The surgical estimated blood loss was reported to be 2,300 cc and the quantitative blood loss was 1,200 cc.

## Outcome

Two vigorous male infants were successfully delivered. Twin A had Apgar scores of 7 and 8 at 1 and 5 minutes, respectively. However, he subsequently experienced respiratory distress requiring continuous positive airway pressure for 12 hours in the neonatal intensive care unit. Twin B had Apgar scores of 8 and 9 at 1 and 5 minutes, respectively. Twin A weighed 2,430 g and twin B weighed 3,070 g.

The patient's postpartum course was first notable for continued elevated creatinine (1.21–1.10 mg/dL prior to delivery), which downtrended over the course of 24 hours. She then experienced worsening abdominal pain and tenderness on a postoperative day 1 with signs of sepsis (tachycardia/hypotension/fever) and a positive focused assessment with sonography for trauma (FAST) scan (6.4 × 3.9 pocket in the right upper quadrant), consistent with a small degree of intrabdominal blood. Due to the concern for sepsis in the setting of clinical signs consistent with endometritis, the patient was treated with cefepime and clindamycin. The patient's blood pressure was responsive to intravenous fluid resuscitation. She also experienced symptomatic anemia, with a hemoglobin drop of 12.2 to 7.8 g/dL, prompting the administration of an additional unit of packed red blood cells. She was discharged on postoperative day 4 after meeting all clinical milestones.

## Discussion

This case report describes a rare occurrence of a spontaneous dichorionic diamniotic twin pregnancy in each uterine horn of a didelphys uterus. The patient, in this case, was a 35-year-old G4P1112 with a history notable for unilateral renal agenesis and prior pregnancies with a didelphys uterus. To our knowledge, this is the first case report on the management of a patient with a dichorionic diamniotic twin pregnancy in a didelphys uterus with concomitant congenital unilateral renal agenesis.

A didelphys uterus is an MDA caused by the failure of Müllerian duct fusion during embryogenesis and is characterized by complete duplication of the uterus and cervix and division of the vagina by a septum. 6 When the vaginal septum is oblique, it may cause obstruction to one vaginal canal, leading to blockage of menstrual flow. MDAs are also commonly associated with Wolffian duct abnormalities, manifesting as renal and urological anomalies. As such, renal abnormalities may be seen in 15 to 30% of nonobstructive uterus didelphys cases, with unilateral renal agenesis being the most common renal abnormality in patients with MDAs. 3 7 8 9 In very rare cases, patients may present with the triad of uterus didelphys, ipsilateral renal agenesis, and an obstructed hemivagina, known as obstructed hemivagina and ipsilateral renal agenesis (OHVIA) syndrome (formerly known as Herlyn–Werner–Wunderlich syndrome). 10 11 While our patient had congenital unilateral renal agenesis, there was a low concern for OHVIA syndrome at the time of diagnosis given the longitudinal, nonobstructive vaginal septum on physical exam. However, prompt diagnosis of this syndrome during adolescence is imperative to prevent long-term complications such as endometriosis, infertility, pelvic inflammatory disease, and adverse pregnancy outcomes. 11 12

Diagnosing uterus didelphys presents challenges due to its frequently asymptomatic nature. This condition can be identified during regular pelvic examinations and verified using techniques such as ultrasound, sonohysterogram, magnetic resonance imaging, and hysterosalpingography. 13 14 Given the association of MDAs with renal anomalies, female patients with prenatally detected renal anomalies should also be screened for MDAs. 11 Our case is a clear example of the benefits of early, prenatal detection of MDAs and associated renal anomalies through prenatal imaging and counseling. Our patient received specialized prenatal care and frequent follow-up visits because the team was previously made aware of her diagnosis. Additional renal function surveillance, in this case, included repeat testing of creatinine levels in the third trimester, a prompt preeclampsia rule-out in the third trimester, and appropriate management of elevated creatinine levels in the late third trimester and early postpartum periods.

Timely diagnosis of uterus didelphys additionally enables tailored management strategies that can mitigate pregnancy and fetal risks associated with uterine anomalies, including miscarriage, fetal growth restriction, preterm labor, preterm premature rupture of membranes, malpresentation, antenatal hemorrhage or abnormal placentation, and stillbirth. 9 15 One study found that fetal growth restriction occurred in 11% of deliveries and preterm labor occurred in 24% of deliveries in women with a didelphys uterus. 9 Wang et al found approximately three times increased odds of fetal growth restriction and preterm labor in deliveries with MDAs. 16 To screen for this increased risk of fetal growth restriction, our team performed serial growth ultrasounds throughout the patient's pregnancy. Our team additionally assessed this patient's risk of preterm labor with serial cervical length measurements up until 22 weeks gestational age.

Cesarean deliveries are more common than spontaneous vaginal deliveries in patients with didelphys uterus, with an occurrence of around 80%. 9 16 However, a twin pregnancy in a uterus didelphys does not always necessitate delivery by cesarean section, unless the pregnancy is complicated by fetal malpresentation, fetal distress, or vaginal obstruction. 9 17 As such, there are reports of both vaginal and cesarean deliveries in dicavitary twin pregnancies. 17 Although fewer case reports exist regarding vaginal delivery in dicavitary twin pregnancies, several successful vaginal deliveries have been documented. 2 18 These reports emphasize that uterus didelphys is not a direct contraindication for spontaneous vaginal delivery. Coordination between the prenatal care provider, the delivery team, and the patient is crucial for ensuring a safe and thoughtful approach to delivery. Given the increased risk of obstetric complications associated with a didelphys uterus, effective collaboration is essential. 19 Through adopting a multidisciplinary approach, healthcare providers can make informed decisions tailored to the specific needs of the patient, ultimately reducing maternal and neonatal morbidity and mortality.

While the fetuses in this case had cephalic presentation, cesarean section was indicated in the setting of multiple prior cesarean deliveries. After the initial Pfannenstiel incision and the first transverse hysterotomy, a vertical hysterotomy on the left uterine corpus was required because of the narrowed lower uterine segment. This approach is similar to another case that reported the use of a transverse incision in the first lower uterine segment and a classical incision in the second lower uterine segment of the second uterus due to a narrow uterine segment. 17 By contrast, another case study reported the use of a single uterine incision to deliver a dicavitary twin pregnancy. 20 More research is needed to identify the optimal mode of delivery for pregnant individuals with didelphys uterus with pregnancy in each uterine cavity.

This patient's postpartum course was complicated by postpartum hemorrhage and endometritis. A retrospective cohort study by Wang et al found no increased risk of postpartum hemorrhage in deliveries of women with MDAs, compared with those without MDAs. 16 However, a cross-sectional study by Mandelbaum et al found an increased risk of hemorrhage in patients with uterus didelphys. 21 Other case studies of singleton and dicavitary didelphys twin pregnancies have also reported both intraamniotic infection and postpartum hemorrhage. 22 23 Given the data presented in the literature, it is feasible that our patient's postpartum complications were related to her diagnosis of didelphys uterus. This highlights the continued risk of postpartum complications in the presence of uterine anomalies, even when diagnosed promptly, and the need for comprehensive postnatal care and ongoing multidisciplinary support.

## Learning Points

- Cesarean delivery is more common in dicavitary twin pregnancies in uterus didelphys, though vaginal delivery may be possible depending on fetal presentation and other factors.
- MDAs can be linked with renal anomalies such as unilateral renal agenesis, as well as other Wolffian duct abnormalities. Patients with prenatally detected renal anomalies should be screened for uterine anomalies.
- Didelphys uterus can present asymptomatically, making diagnosis challenging. Early detection allows for tailored management strategies, including frequent follow-ups and monitoring for adverse outcomes such as preterm delivery and fetal growth restriction.
- Patients undergoing cesarean section for dicavitary twin pregnancy in didelphys uterus are at an increased risk of postpartum hemorrhage, not only due to uterine atony but also as a result of bleeding at the hysterotomy site.
- Despite early diagnosis, patients may still face complications such as peripartum hemorrhage and endometriosis, emphasizing the need for vigilant predelivery planning to ensure preparedness for these at-risk intrapartum complications.
